# RNR2 repression by p21 restricts reverse transcription of HIV-1 and related-lentiviruses in macrophages

**DOI:** 10.1186/1742-4690-10-S1-O27

**Published:** 2013-09-19

**Authors:** Awatef Allouch, Annie David, Sarah Amie, Hichem Lahouassa, Loic Chartier, Florence Margottin-Goguet, Françoise Barré-Sinoussi, Baek Kim, Asier Saez-Cirion, Gianfranco Pancino

**Affiliations:** 1 Unite de Regulation des Infections Rétrovirales, Institut Pasteur de Paris, Paris, France; 2 Department of Microbiology and Immunology, University of Rochester Medical Center, Rochester, New York, USA; 3 INSERM, U1016, Institut Cochin, 27 Rue du Faubourg St Jacgues, Bat G. Roussy, Paris, France; 4 Unité de Recherche et d’Fxpertise Epidémiologie des Maladies Emergentes Institut Pasteur de Paris, Paris, France; 5 CNRS, UMR8104, Paris, France; 6 INSERM, Paris, France

## Background

Macrophages play crucial roles in HIV/AIDS pathogenesis as they are important targets for HIV-1 replication and contribute to viral spread and viral reservoir formation. We have previously reported that p21 inhibits HIV-1, HIV-2 and SIV replication in macrophages by a major block at the level of reverse transcription [1]. This study aimed at understanding the molecular mechanisms involved in p21-mediated restriction.

## Materials and methods

Monocyte-derived macrophages (MDM) were differentiated from monocytes isolated from healthy donor blood with human AB serum.The induction of p21 expression levels was obtained by the stimulation of MDM by the immobilized immunoglobulins (IVIg). MDM were infected with NL4.3-Luc HIV-1 pseudotyped with VSV-G or replication-competent SIVmac251. HIV-1 and SIV late reverse transcripts were quantified by Taqman quantitative real-time PCR (qRT-PCR). p21, RNR2, E2F1 and RB knockdowns were obtained by small interfering RNAs (siRNAs) transfection of MDM. Silencing efficiencies were determined by qRT-PCR mRNA quantification and by western blot. Intracellular dNTP concentrations were measured by a single-nucleotide incorporation assay. Statistical analyses were performed with the Kruskal-Wallis and the Mann-Whitney tests.

## Results

Through overexpression and knockdown experiments in primary monocyte derived macrophages, we demonstrated that p21 inhibits HIV-1 reverse transcription by down-regulating the intracellular dATP and dTTP pools available for viral cDNA synthesis. We found that p21 represses the expression of ribonucleotide reductase subunit R2 (RNR2), an enzyme indispensable for dNTP biosynthesis. Accordingly, depletion of RNR2 either by p21 induction or RNR2-specific siRNA transfection inhibits reverse transcription of HIV-1 in macrophages. Noteworthy, p21 induction and RNR2 knockdown also restrict SIVmac251 in macrophages, in spite of Vpx-mediated degradation of SAMHD1.

We further explored the mechanisms underlying the inhibition of RNR2 expression by p21 and found that p21 represses the expression of the RNR2 transcriptional transactivator E2F1, independently from the Retinoblastoma protein RB. Consistently, the depletion of E2F1 by p21 induction or E2F1-specific siRNA transfection represses RNR2 transcription and inhibits HIV-1 reverse transcription in macrophages.

## Conclusion

Here we describe a new mechanism of restriction of lentiviral replication in macrophages: p21 inhibits HIV-1 reverse transcription by affecting dNTP biosynthesis through repression of RNR2 expression differently from SAMHD1 restriction that promotes dNTP catabolism. Accordingly, p21 restricts other primate lentiviruses able to escape SAMHD1 restriction through Vpx, such as HIV-2 and SIV Our findings describe a novel metabolic pathway of HIV-1 restriction in macrophages and open on new potential cellular targets for therapy research.
